# The effect of heart failure and left ventricular assist device treatment on right ventricular mechanics: a computational study

**DOI:** 10.1186/s12938-018-0498-0

**Published:** 2018-05-22

**Authors:** Jun I. K. Park, Aulia Khamas Heikhmakhtiar, Chang Hyun Kim, Yoo Seok Kim, Seong Wook Choi, Kwang Soup Song, Ki Moo Lim

**Affiliations:** 1 Department of IT Convergence Engineering, Kumoh National Institute of Technology, 61 Daehak-ro, Gumi, Gyeongbuk 39177 Republic of Korea; 2 Department of Mechanical & Biomedical Engineering, Kangwon National University, Kangwon, Republic of Korea; 3 Department of Medical IT Convergence Engineering, Kumoh National Institute of Technology, Gumi, Republic of Korea

**Keywords:** Heart failure, Left ventricular assist device, Electromechanical model, Right ventricle

## Abstract

**Background and aims:**

Although it is important to analyze the hemodynamic factors related to the right ventricle (RV) after left ventricular assist device (LVAD) implantation, previous studies have focused only on the alteration of the ventricular shape and lack quantitative analysis of the various hemodynamic parameters. Therefore, we quantitatively analyzed various hemodynamic parameters related to the RV under normal, heart failure (HF), and HF incorporated with continuous flow LVAD therapy by using a computational model.

**Methods:**

In this study, we combined a three-dimensional finite element electromechanical model of ventricles, which is based on human ventricular morphology captured by magnetic resonance imaging (MRI) with a lumped model of the circulatory system and continuous flow LVAD function in order to construct an integrated model of an LVAD implanted-cardiovascular system. To induce systolic dysfunction, the magnitude of the calcium transient function under HF condition was reduced to 70% of the normal value, and the time constant was reduced by 30% of the normal value.

**Results:**

Under the HF condition, the left ventricular end systolic pressure decreased, the left ventricular end diastolic pressure increased, and the pressure in the right atrium (RA), RV, and pulmonary artery (PA) increased compared with the normal condition. The LVAD therapy decreased the end-systolic pressure of the LV by 41%, RA by 29%, RV by 53%, and PA by 71%, but increased the right ventricular ejection fraction by 52% and cardiac output by 40%, while the stroke work was reduced by 67% compared with the HF condition without LVAD. The end-systolic ventricular tension and strain decreased with the LVAD treatment.

**Conclusion:**

LVAD enhances CO and mechanical unloading of the LV as well as those of the RV and prevents pulmonary hypertension which can be induced by HF.

## Background

In left heart failure (LHF), pulmonary arterial hypertension is likely to occur and result in right ventricular hypertrophy, which alter the contractility of the right ventricle (RV). In addition, as the cardiac output (CO) decreases, other complications are likely to occur, including tricuspid regurgitation, hypertension in the hepatic venous causing hepatic dysfunction, and coronary ischemia due to the abnormal circulation in the coronary system [1].

In LHF, left ventricular assist device (LVAD) implantation reduces left ventricular workload and increases CO. The increased CO reduces right ventricular workload and pulmonary blood pressure. However, alteration of ventricular septal position owing to LVAD implantation is likely to impair the contractility of the RV and cause tricuspid regurgitation [1–3].

There are several studies on risk scores for stratification associated with right ventricular dysfunction after LVAD implantation. Grant et al. [4] examined the function of the RV in 117 patients with continuous LVAD implantation and determined that the peak value of the longitudinal strain of the RV free wall, which is a risk score of right ventricular dysfunction, was higher than − 9.6%. Dandel et al. [5] stratified risk scores by measuring echocardiography in order to obtain the effect of afterload on the RV geometry or velocity of contraction.

Although the quantitative analysis of the hemodynamic factors related to the RV after LVAD implantation is critical, previous studies have focused on the alteration of the ventricular shape and lack quantitative analysis of the various hemodynamic parameters. Furthermore, experimental studies consumed more time and have less cost-efficient. There are also various limitations of experimental study as it is likely to invasively harm an actual human heart. Simulation studies, on the other hand, can overcome those limitations and provide data on a variety of hemodynamic parameters in a noninvasive manner.

In this study, various hemodynamic parameters related to the RV were quantitatively analyzed under normal, heart failure (HF), and HF incorporated with continuous flow LVAD conditions by using a computational model [6–10]. The simulation was conducted with an ideal case which represents no morphological influence to the geometric structure of the ventricles despite of LVAD implantation and involved a three dimensional finite element electromechanical model of human ventricles coupled with a lumped model of the circulatory system [11–13].

## Methods

**Fig. 1:**
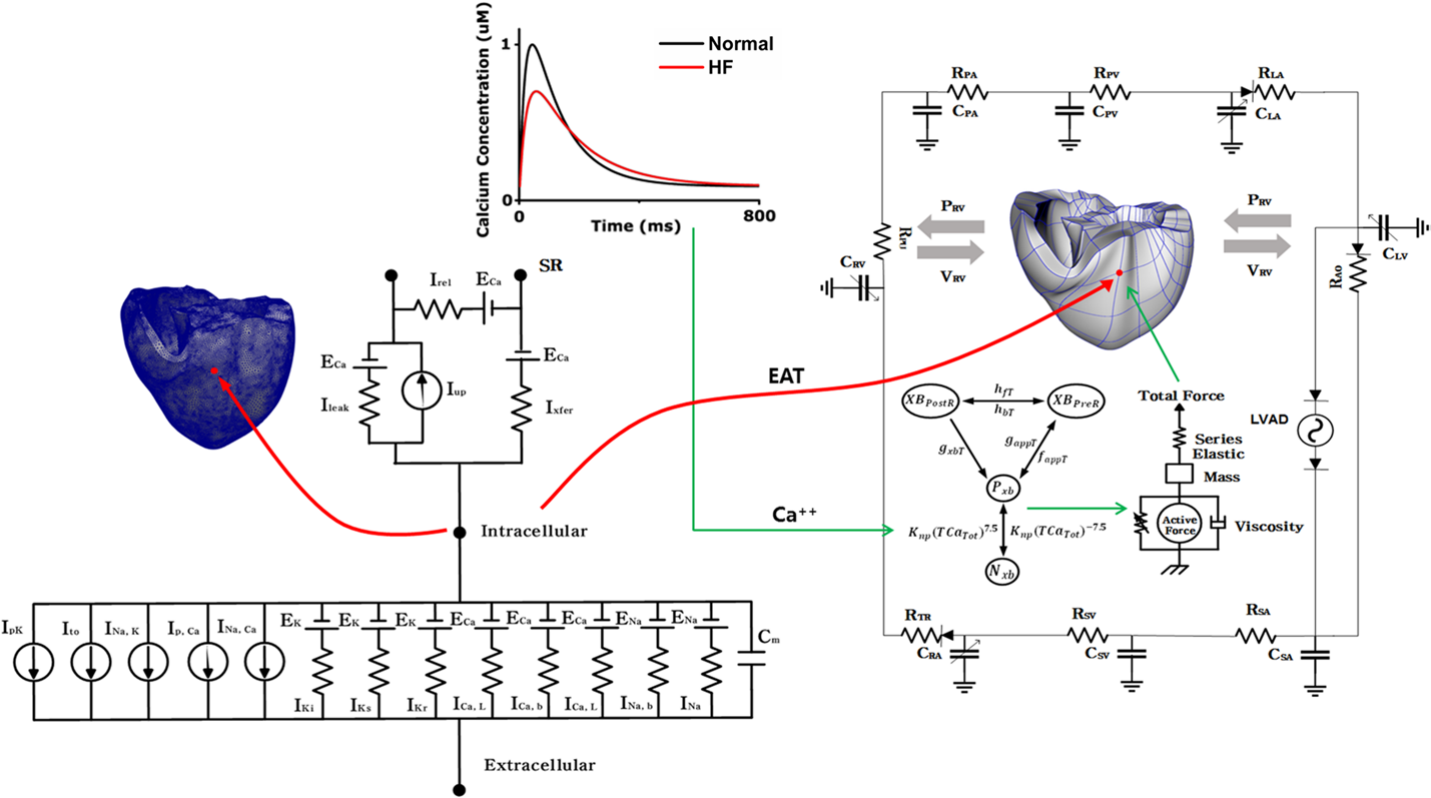
Schematic diagram of the electrical and mechanical finite-element ventricular model coupled with calcium transient, circulatory system, and continuous flow LVAD models. Electrical model: fast inward Na^+^ current (I_Na_), background Na^+^ current (I_Na, b_), L-type inward Ca^2+^ current (I_Ca, L_), background Ca^2+^ current (I_Ca, b_), rapid delayed rectifier K^+^ current (I_Kr_), slow delayed rectifier K^+^ current (I_Ks_), inward rectifier K1 current (I_K1_), Na^+^–Ca^2+^ exchange current (I_Na, Ca_), sarcoplasmic Ca^2+^ pump current (I_p, Ca_), Na^+^–K^+^ exchange current (I_Na, K_), transient outward K^+^ current (I_to_), K^+^ pump current (I_p, K_), Ca^2+^ release current from the JSR (I_rel_), Ca^2+^ leak current from the JSR (I_rel_), and Ca^2+^ uptake current into the NSR (I_up_). *EAT* electrical activation time. Mechanical element: *PRV* RV pressure, *VRV* RV volume, *PLV* LV pressure, *VLV* LV volume, *RPA* pulmonary artery resistance, *CPA* pulmonary artery compliance, *RPV* pulmonary vein resistance, *CPV* pulmonary vein compliance, *RMI* mitral valve resistance, *CLA* left atrium compliance, *RAO* aortic valve resistance, *RSA* systemic artery resistance, *CSA* systemic artery compliance, *RSV* systemic vein resistance, *CSV* systemic vein compliance, *RTR* tricuspid valve resistance, *CRA* right atrium compliance, *RPU* pulmonary valve resistance. The section under the mechanical model, which receives the calcium as input, reveals the calcium and cross-bridge activation status [8]. *Nxb* nonpermissive confirmations of the regulatory proteins, *Pxb* permissive confirmations of the regulatory proteins, *XBpreR* transition of pre-rotated, which is the binding of myosin head to the actin, *XBpostR* post-rotated state

The mathematical model used to carry out this study consists largely of four components. First is an electrical conduction model of ventricular tissue. Second is electrical conduction model of Purkinje fiber, which is a pathway to transmit electrical signal to ventricular tissue. Third is a mechanical contraction model of ventricles. Fourth is a lumped parameter model of atrial function and vascular system, which constitute a whole cardiovascular system model with a ventricular mechanical model. Figure 1 shows a schematic of the whole cardiovascular system model.

We used a previously reconstructed human ventricular model based on publicly available MR imaging with both fiber orientation information and cardiac tissue inhomogeneity information [14, 15]. The procedure is, first, myocardium is separated from the suspension media by doing level-set segmentation on the MR image stacks. Second, the ventricles are segmented from the atria. Third, for every tenth slice within the MR image stack, landmark points are manually seeded to identify the atrioventricular border, which are determined by the location of the valves and gray level differences. Fourth, a 3D cubic Hermite is fitted through the landmark points to generate a surface that represents the atrioventricular border. The surface mesh serves as a guide for the creation of the finite element mesh of the heart model. Fiber and laminar sheet structural information for the ventricles is obtained from the diffusion tensor magnetic resonance (DTMR) image data set. Two dimensional Purkinje network geometry of Berenfeld and Jalife [16]. was then mapped onto the three dimensional endocardial surface of the ventricle model [17].

### Electrical model

The mathematical description of electrical conduction is governed by the monodomain representation of cardiac tissue. A membrane kinetics model represented the electrical activity at the cellular level. We used the membrane dynamic model of ten Tusscher as it was originally formulated [18]. The governing equations of electrical conduction through three-dimensional ventricle tissue are the following partial differential equations in reaction–diffusion from [19]:

1 $$\nabla \cdot \tilde{\sigma }\nabla V_{m} \, = \, \beta I_{m}$$

2 $$I_{m} \, = \,C_{m } \frac{{\partial V_{m} }}{\partial t}\, + \,I_{ion} (V_{m, } v)\, - \,I_{trans}$$

where $\tilde{\sigma }$ is the intracellular conductivity tensor, *β* is the surface-to-volume ratio of cardiac cells, *I*_*trans*_ is the current density of the transmembrane stimulus, *C*_*m*_ is the membrane capacitance per unit area, *V*_*m*_ is the membrane potential, and *I*_*ion*_ is the current density of the ionic current, which depends on the transmembrane potential and other state variables represented by *υ*. *I*_*ion*_ is the sum of all transmembrane ionic currents given by the following equation [18]

3 $$\begin{aligned} I_{ion} \, =& \,I_{Na} \, + \, I_{K1} \, + \, I_{to} \, + \, I_{Kr} \, + \, I_{Ks} \, + \, I_{CaL} \, + \,I_{NaCa} \hfill \\ \quad &+ \, I_{NaK} \, + \,I_{pCa} \, + \,I_{pK} \, + \,I_{bCa} \, + \,I_{bNa} . \hfill \\ \end{aligned}$$

For the electrophysiological simulation of the ventricles, finite tetrahedral linear elements were generated using HyperMesh software (214,319 nodes and 1,061,379 elements). An electrical impulse is generated at the AV node at the top of the Purkinje network and conduction occurs along the Purkinje network. Conduction through the Purkinje fibers maintained a clinical conduction speed of 200 cm/s. When the impulse reaches the 145 Purkinje terminal nodes at approximately the same time, it excites the ventricular tissue at the Purkinje terminal node, resulting in electrical conduction throughout the ventricles.

To solve the governing equations, we used a forward Euler method with a time step of 0.01 ms for temporal discretization, and the Galerkin approach of the finite element method for spatial discretization. Solving the governing equations in this way will reveal all of the dependent variables included in the governing equations. The intracellular calcium concentration transients calculated at all of the discretized nodes are used as input to the cardiac mechanical contraction model.

### Mechanical model

The mathematical description of the mechanical contraction of cardiac tissue is based on continuum mechanics [20, 21], with myocardium assumed to be a hyperelastic, nearly incompressible material, the passive mechanical properties of which were defined using an exponential strain function [22].

4 $$W\, = \,\frac{\text{C}}{2}\left( {e^{Q} \, - \,1} \right)$$

5 $${\text{Q}}\, = \,b_{1} E_{ff}^{2} + b_{2} \left( {E_{rr}^{2} \, + \,E_{cc}^{2} \, + \,2E_{rc}^{2} } \right)\, + \,2b_{3} \left( {E_{fr}^{2} \, + \,E_{fc}^{2} } \right)$$

where W is a strain energy function, and the Green–Lagrange strains Eij are referred to the local fiber coordinate system. C = 2 kPa, b_f_ = 8, b_t_ = 2, and b_fs_ = 4. The laminar sheet and fiber orientation information determined the orthotropic electrical conductivity and passive mechanical properties of the myocardium. A finite element mesh consisting of 462 nodes and 230 Hermite elements was used. A cross-bridge dynamics model [23] represented the generation of active tension at the level of a single myocyte.

To simulate hemodynamic responses, which are the interactions between the blood and ventricles, the finite element electromechanical model of the human ventricle was coupled with a circulatory model using the coupling method of Gurev et al. [24, 25]. A schematic diagram of the integrated model is illustrated in Fig. 1.

Physiologically, depolarization of each myocyte occurs by electrical wave propagation of the heart. Depolarization of the myocyte activates the calcium channel to release calcium from the sarcoplasmic reticulum into the cytosol; this causes the calcium to bind to the troponin C followed by cross-bridge contraction cycle due to sliding of the myofilaments. The cross-bridge contraction cycling forms the basis for contractile protein movement and development of active tension in the myocyte, resulting in deformation of the ventricles. The process by which the calcium acts as the actuator to convert electrical activation into mechanical phenomenon was implemented by inputting the intracellular calcium concentration obtained from the electrophysiological model from each myocardial cell into the contractile myofilament dynamics model (see Fig. 1).

### LVAD model

The finite element electromechanical model of the human ventricle was combined with a lumped model of the circulatory system, cardiovascular system, and continuous flow LVAD model (Fig. 1). The continuous LVAD was connected to the electromechanical and circulatory models through an inlet in the left ventricle, and the outlet was attached to the aorta in the integrated circulatory model. In this study, continuous flow LVAD was used because it exhibited higher efficiency, size, implantability, extended support, and overall patient outcomes compared to pulsatile LVAD [26–29]. The LVAD type was modeled as a flow generator, and a specific flow rate of 4 L/min was set for the continuous LVAD.

### Simulation protocol

**Fig. 2:**
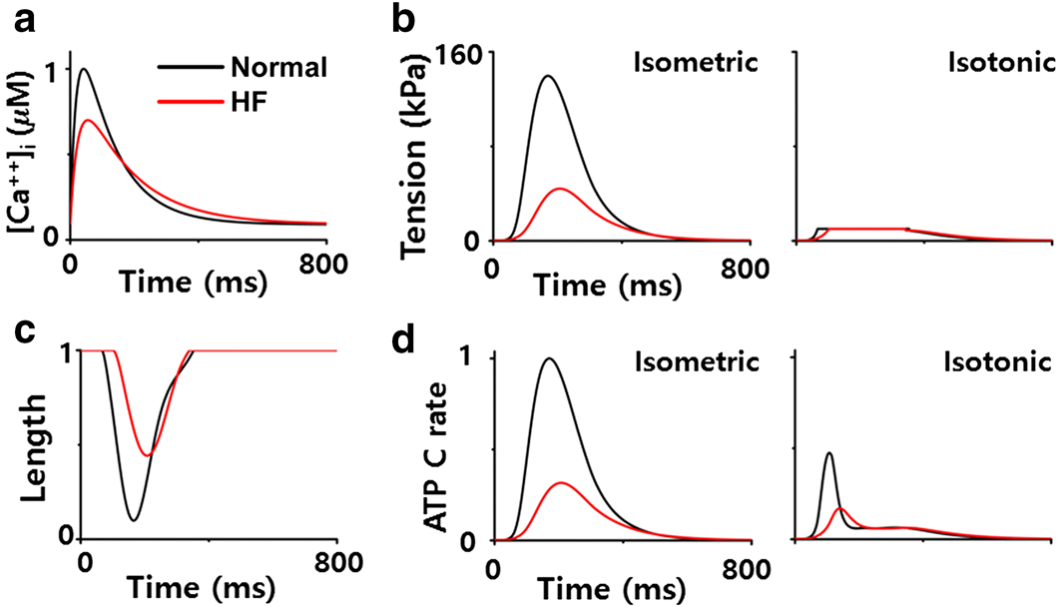
Results of mechanical simulation of single cell under normal (Ori) and HF conditions. **a** Intracellular calcium transient Calcium concentration, **b** myocardial tension, **c** muscle shortening, and **d** ATP consumption rate as obtained from simulations under isotonic (load = 10 kPa) and isometric (load = 1000 kPa) conditions with Ori and HF cases during one cycle. The calcium transient is normalized by the peak calcium concentration under normal conditions. Muscle length is normalized by its value in the isotonic contraction phase. The ATP consumption rate is normalized by the peak ATP consumption rate under Ori and isometric conditions

Single cell simulation: The basic cycle length (BCL) was set as 800 ms, and the calcium transient scaling factor of HF was set as 0.7. In this study, 10 cycles were simulated under each of isometric and isotonic conditions, and the data was gained from last one cycle to observe changes on steady state (Fig. 2).

Three dimensional electromechanical simulation: A total of 25 cycles were simulated to attain a steady state (BCL = 800 ms). After electrical simulation, the EAT data as well as the calcium concentration data of normal and HF myocardial cells (Fig. 2a) were collected and they were entered as inputs to the mechanical simulation. We compared hemodynamic parameters of the last one cycle under normal, HF, and HF with continuous LVAD conditions.

## Results

Three cases were simulated under normal, HF, and HF with continuous LVAD conditions. We analyzed the mechanical responses which were results of simulation, including the pressures of the left ventricle (LV), aorta, right atrium (RA), right ventricle (RV), and pulmonary artery (PA); biventricular pressure–volume diagram; quantitative hemodynamic responses provided in Table 1; flow rate through tricuspid and pulmonary valves; and transmural distribution of tension and strain.

**Table 1:** Quantitative data of hemodynamic parameters of LV and RV

	Normal	HF	HF LVAD
LVESP (mmHg)	110	73	43
LVEDP (mmHg)	5	12	− 1
LVPP (mmHg)	105	61	44
RAESP (mmHg)	4	7	5
RVESP (mmHg)	16	19	7
RVEDP (mmHg)	1	5	2
PAESP (mmHg)	13	17	5
RVPP (mmHg)	15	14	5
LVCO (L/min)	3.8	2.4	1.5
LVEDV (mL)	112	117	89
LVESV (mL)	61	85	69
LVSV (mL)	51	32	20
LVEF (%)	46	27	22
LVSW (mmHg mL)	4610	1673	446
RVCO (L/min)	3.8	2.5	3.5
RVEDV (mL)	136	157	144
RVESV (mL)	85	124	98
RVSV (mL)	51	33	46
RVEF (%)	38	21	32
RVSW (mmHg mL)	648	411	137

*LVESP* left ventricular end systolic pressure, *LVEDP* left ventricular end diastolic pressure, *LVPP* left ventricular pulse pressure, *RAESP* right atrial end systolic pressure, *RVESP* right ventricular end systolic pressure, *RVEDP* right ventricular end diastolic pressure, *PAESP* pulmonary arterial end systolic pressure, *RVPP* right ventricular pulse pressure, *LVCO* left ventricular cardiac output, *LVEDV* left ventricular end diastolic volume, *LVESV* left ventricular end systolic volume, *LVSV* left ventricular stroke volume, *LVEF* left ventricular ejection fraction, *LVSW* left ventricular stroke work, *RVCO* right ventricular cardiac output, *RVEDV* right ventricular end diastolic volume, *RVESV* right ventricular end systolic volume, *RVSV* right ventricular stroke volume, *RVEF* right ventricular ejection fraction, *RVSW* right ventricular stroke work

**Fig. 3:**
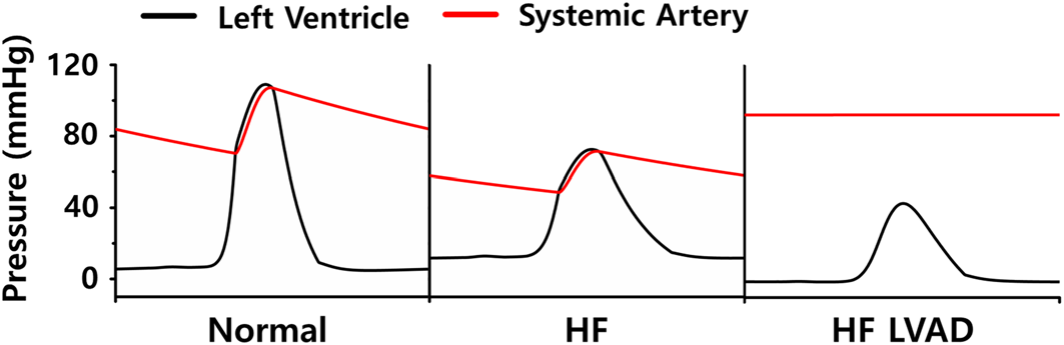
Pressures of left ventricle and systemic artery under normal, HF, and HF incorporated with continuous flow LVAD conditions

Figure 3 illustrates the pressure in the LV and aorta (systemic artery) at the last one cycle of a steady state responses (BCL = 800 ms). Under HF condition, the left ventricular end systolic pressure (LVESP) decreased from 110 mmHg (normal) to 73 mmHg (HF), and the left ventricular end diastolic pressure (LVEDP) increased from 5 to 12 mmHg. When the continuous LVAD was applied, LVESP decreased from 73 mmHg (HF) to 43 mmHg (HF LVAD), LVEDP decreased from 12 mmHg to − 1 mmHg (minus pressure denotes below air pressure), and left ventricular pulse pressure decreased from 61 to 44 mmHg.

**Fig. 4:**
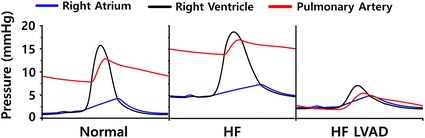
Pressures of right atrium, right ventricle, and pulmonary artery under normal, HF, and HF incorporated with continuous flow LVAD conditions

Figure 4 illustrates the pressure in the RA, RV, and PA at the last one cycle of a steady state responses (BCL = 800 ms). Under HF condition, the pressure in the RA, RV, and PA is higher than those under normal condition, and when the LVAD was applied, the pressures decreased. The right atrial end systolic pressure (RAESP) decreased from 7 mmHg (HF) to 5 mmHg (HF LVAD), the right ventricular end systolic pressure (RVESP) decreased from 19 to 7 mmHg, right ventricular end systolic pressure (RVEDP) decreased from 5 to 2 mmHg, and the pulmonary arterial end systolic pressure (PAESP) decreased from 17 to 5 mmHg. The right ventricular pulse pressure (RVPP) decreased from 14 mmHg (HF) to 5 mmHg (HF LVAD) after LVAD implantation. Under HF condition, the RV pressure was higher than that under normal condition; however, when the LVAD was applied, the RV pressure decreased.

**Fig. 5:**
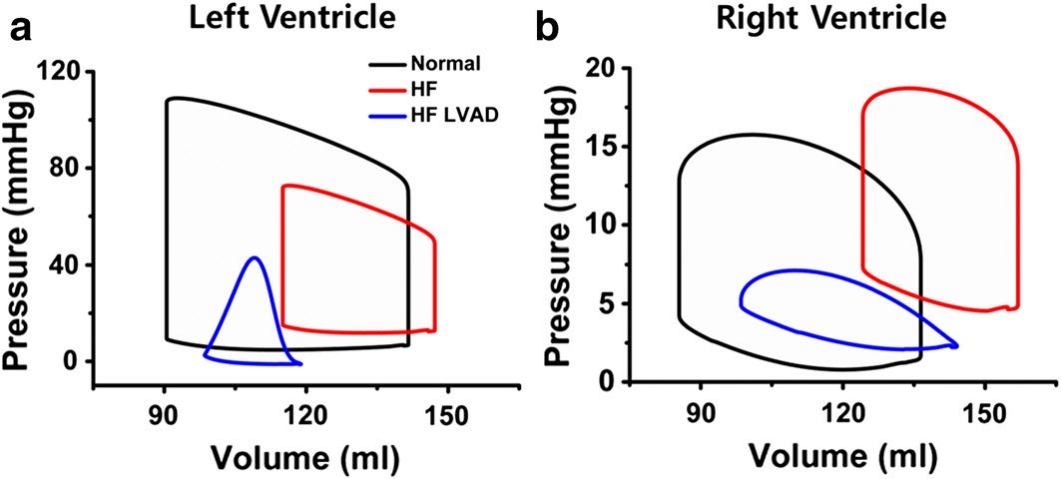
Pressure–volume loops of left ventricle **a** and right ventricle **b**

Figure 5a illustrates the pressure–volume (PV) loops of the LV under normal, HF, and HF with LVAD conditions. In HF without LVAD, the LV–PV loop was shifted to the right compared to the normal condition. The left ventricular end diastolic volume (LVEDV) increased from 112 to 117 mL, left ventricular end systolic volume (LVESV) increased from 61 to 85 mL, and left ventricular stroke volume (LVSV) decreased from 51 to 32 mL. In a manner similar to that of the results in Fig. 3, the LVESP decreased, LVEDP increased, and left ventricular stroke work (LVSW) which is denoted by the internal area of the PV loop decreased from 4610 to 1673 mmHg mL.

When the LVAD is applied under HF, the LVESV and LVEDV decreased from 85 to 69 mL and from 117 to 89 mL, respectively, and no isovolumetric contraction and relaxation were observed. In addition, the LVSV decreased from 32 to 20 mL, and the pressure also decreased in a manner to that of the results in Fig. 3. The LVSW decreased from 1673 to 446 mmHg mL.

Figure 5b illustrates the PV loops of the RV. In HF condition, the PV loop of the RV was shifted to the right as compared to normal condition; moreover, the right ventricular diastolic volume (RVEDV) increased from 137 to 157 mL, right ventricular systolic volume (RVESV) increased from 85 to 124 mL, right ventricular cardiac output (RVCO) decreased from 3.8 to 2.5 L/min, and RVPP decreased in a manner to that of the results in Fig. 4.

In HF with LVAD, the isovolumetric alterations were more or less eliminated. The RVESV and RVEDV decreased from 124 to 98 mL and from 157 to 144 mL, respectively. In addition, the right ventricular stroke volume (RVSV), which is the difference between the RVESV and RVEDV, increased from 33 to 46 mL, and the right ventricular stroke work (RVSW) decreased from 411 to 137 mmHg mL.

**Fig. 6:**
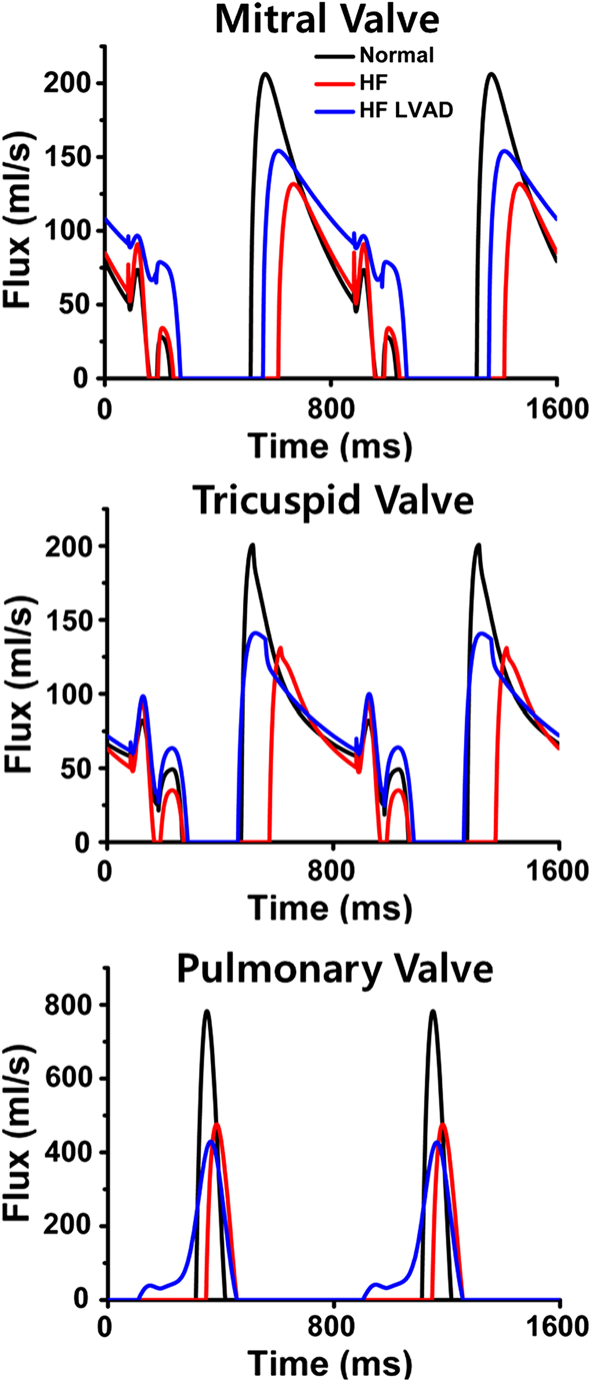
Fluxes of mitral valve, tricuspid valve, and pulmonary valve under normal, HF, and HF incorporated with continuous flow LVAD conditions during two cycles

Figure 6 illustrates the fluxes (flow rates) through the mitral valve (MV), tricuspid valve (TV), and pulmonary valve (PV) during two cycles (1600 ms) under normal, HF, and HF with the LVAD conditions. In the case of HF, the flux through MV, TV, and PV are lower, and the duration of valvular opening are shorter compared to normal condition; consequently, the blood flow into and out of the ventricles decreased. This phenomenon is similar to that depicted by Fig. 5, wherein the RVSV and RVCO are lower under HF condition. LVAD treatment increased the flux through the MV, TV, and PV as well as their opening duration compared to HF condition. These results are similar to that in Fig. 5, wherein the RVSV and RVCO with LVAD treatment are higher.

**Fig. 7:**
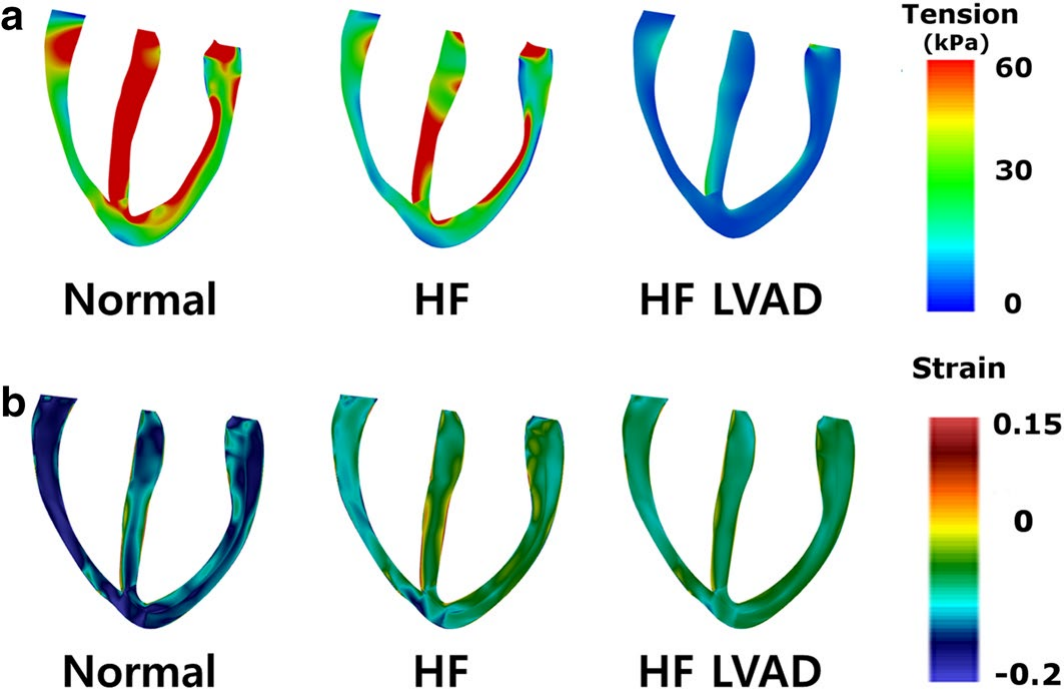
Transmural distribution of the ventricular tension **a** and strain **b** at the end-systole under normal, HF, and HF incorporated with continuous flow LVAD conditions

Figure 7a illustrates the transmural distribution of the ventricular tension at end-systole under normal, HF, and HF with LVAD conditions. Actively generated tension was lower in HF compared to normal condition. LVAD treatment decreased the ventricular tension further.

Figure 7b illustrates the transmural distribution of the ventricular strain at end-systole under normal, HF, and HF with LVAD conditions. In case of HF, the negative strain decreased compared to that in normal condition. When the LVAD was applied, the negative strain also decreased compared to the condition without LVAD. A positive value of ventricular strain signifies that the ventricles expand during ventricular dilatation, and their volume increases from that in resting state. A negative value signifies that the ventricles contract during contraction, and their volume decreases from that in resting state.

## Discussion

In this study, we quantitatively analyzed various hemodynamic parameters related to the RV under the ideal case that represents no morphological influence to the geometric structure of ventricles despite of LVAD implantation through computational simulation. The main findings of this study were the following:

1. Under HF condition, the LVESP decreased and the LVEDP increased and the pressure in the RA, RV and PA also increased compared to the normal condition. When the continuous LVAD was applied, pressure in the LV, RA, RV and PA decreased (Figs. 3, 4, Table 1).
2. The biventricular PV loops in HF condition were shifted to the right side compared to the normal condition. When the LVAD was applied, the biventricular PV loops were shifted to the left side and the LVSV decreased but RVSV increased (Fig. 5, Table 1).
3. Under HF condition, the fluxes (flow rates) through the MV, TV, and PV were lower and the duration of opening valve shorter compared to normal condition. But LVAD treatment increased the fluxes through the MV, TV and PV and the duration of opening valve (Fig. 6).
4. In case of HF, the end systolic ventricular contraction force and strain decreased from that in normal condition. When the LVAD was applied, the contraction force and strain also further decreased (Fig. 7).

In the case of HF, the contractile force is weaker (Fig. 2a, b) than normal force because the intracellular concentration of calcium released from sarcoplasmic reticulum was lower than that in normal condition. Therefore, the systolic pressure of the LV and aorta also decreased. As the contraction force had weakened, the diastolic pressure increased with the escalation of the residual blood volume. Under HF condition with the LVAD, the aortic pressure was always higher than the LV pressure regardless of the left ventricular contraction because LVAD continuously pumps blood from the LV to the aorta at 4 L/min. Under HF condition without LVAD, as the CO of the LV is not adequate for normal circulation, the RV preload is marginal, and the RV pressure is higher than that under normal condition. The LVAD treatment increased CO and blood flow to the RV (preload) and reduced the pressures in the RA, RV, and PA (afterload). These results are consistent with the clinical results of Argiriou et al. [1] (Figs. 3, 4).

Owing to the lower ventricular contractility under HF, the intraventricular residual blood volumes were higher; therefore, both the ventricular PV loops were shifted to the right side in HF condition than in normal condition. The LVAD pumps the remnant blood continuously to the aorta such that no isovolumetric contraction and relaxation period was observed. Although the left ventricular CO (LVCO) and ejection fraction (LVEF) decreased from 2.4 to 1.5 L/min and from 27 to 22%, respectively, the practical CO, which is generated by natural LV and LVAD, increased, which resulted in increase of the right ventricular CO (RVCO) and EF (RVEF) from 2.5 to 3.5 L/min and from 21 to 32%, respectively. Furthermore, the fluxes (flow rates) through the MV, TV, and PV also increased (Figs. 5, 6).

Under HF with LVAD implantation condition, the contraction force also decreased because the LVAD decreased the afterload of the ventricles by pumping a partial blood volume inside the ventricular cavity. Therefore, in case of HF, the contraction force decreased from that in normal condition; the negative strain also decreased because of decrease in volume by amount smaller than that in normal condition. Furthermore, in the case of LVAD application, the contraction force was lower than that under no LVAD condition; the negative strain also decreased (Fig. 7).

Previous studies on the effect of the LVAD on the RV had focused only on the right ventricular dysfunction due to the geometric structure of the ventricle associated with the alteration of the interventricular septum. There was a lack of studies to quantitatively analyze the various hemodynamic response associated with the RV, and clinical trials had several limitations. Therefore, this study quantitatively analyzed various hemodynamic responses related to the RV without alterations in ventricular septal position under normal, HF, and HF incorporated with continuous LVAD conditions through simulation using a three dimensional finite element electromechanical model of the human ventricles coupled with a lumped model of the circulatory system [30].

As illustrated in Figs. 3, 4, 5, 6 and 7 and Table 1, the PV loop of the RV was shifted to the right; RVESP and RVEDP increased; and RVPP, RVSV, RVCO, RVEF, and RVSW decreased under HF condition compared to normal condition. When the LVAD was applied, the PV loop of the RV was shifted to the left; RVESP, EVEDP, RVPP, RVSW decreased; and RVCO, RVEF, and RVSV increased; flux of TV and PV increased; and tension and negative strain decreased. It was established that in the absence of unfavorable RV geometry due to alteration of position of interventricular septum after LVAD implantation, LVAD exerted positive effects on the RV because the pressure of the RV and RVSW decreased (pressure unloading), and the RVCO increased.

There are several limitations of the present study. First, experimental or clinical data were not collected as part of this study. Instead, the validated cell model and methodologies from previous studies were applied. For example, the myofilament dynamics model from Rice et al. [23] was applied in order to implement myofilament cross-bridge dynamics. Though the predicted data varied from the data obtained from clinical trials using actual human hearts, there is no significant difference [1, 2]. To reduce modeling complexity, coronary circulation was not implemented. Moreover, one-way EC Coupling model was implemented in this study; consequently, the cardiac mechanical activity could not affect the electrophysiological behavior of the heart, although such phenomena could occur physiologically. Next, the lumped model of the circulatory system was used to reduce the complexity of the model. However, these potential limitations are not expected to influence the conclusion significantly.

## Conclusions

The continuous LVAD enhance CO and mechanical unloading of the LV as well as those of the RV and prevents pulmonary hypertension that can be induced by LHF. With enhanced LVAD technique and surgical skills, there would be no alteration in the position of the ventricular septum after LVAD implantation. Therefore, the effect of continuous LVAD on hemodynamic responses of the RV under ideal case could be predicted through this study.
